# Gingival Depigmentation Using Microneedling Technique With Topical Vitamin C: A Prospective Case Series

**DOI:** 10.7759/cureus.35345

**Published:** 2023-02-23

**Authors:** Diana Mostafa, Nader A.Alaizari, Shaden M.AlOtaibi, Najla Ahmed Aldosari, Jamilah Rabie Al-Anazi, Rawa S.Alsughayer, Hadeel M.AlFayir, Mona S.AlHarthi, Maram H.AlAnazi

**Affiliations:** 1 Departement of Clinical Periodontology, Alexandria University, Alexandria, EGY; 2 Department of Oral Medicine and Diagnostic Sciences, Vision Colleges for Dentistry and Nursing, Riyadh, SAU; 3 Department of Preventive Dental Sciences, Vision Colleges for Dentistry and Nursing, Riyadh, SAU

**Keywords:** dermapen, depigmentation of gingiva, gingiva, topical vitamin c, microneedling technique, gingival depigmentation, gingival hyperpigmentation

## Abstract

Introduction

Gingival pigmentation is mainly physiological due to the production of melanin. This study aimed to evaluate the effectiveness of a microneedling technique using topical ascorbic acid in treating gingival hyperpigmentation.

Methods

A case-series study was established, and 16 out of 42 participants enrolled in this study according to the inclusion and exclusion criteria. A microneedling technique was performed using a Dermapen instrument, followed by the topical application of ascorbic acid on the pigmented gingiva. Variations in the Dummett oral pigmentation index (DOPI) and Hedin melanin index (HMI) scores were considered for each patient. A one-month follow-up was conducted on all patients.

Results

All the reported cases demonstrated noticeable improvement at the end of the sessions. Moreover, seven patients showed complete depigmentation of the gingiva. Analysis using paired T-tests showed a statistically significant lower post-treatment DOPI score with a mean difference of 1.8 ± 0.7, 95% CI: 0.17-1.49. Similarly, the HMI score was lower post-treatment with a mean difference of 3.1 ± 0.7, 95% CI: 2.74-3.50.

Conclusions

Microneedling combined with topical ascorbic acid is a novel, non-invasive dental technique that can effectively treat gingival hyperpigmentation.

## Introduction

Today, smile aesthetics have become a critical element in dentistry to achieve a harmonious appearance of the teeth and gingiva. In terms of overall aesthetics and appearance, the colour of the gingiva plays a vital role, as gingival pigmentation (GP) may cause embarrassment, particularly if it is visible during speech and smiling. As well as treating biological and functional problems, the periodontist has to achieve satisfactory gingival aesthetics. The gingival tissues are normally pale pink, but some people have gingival melanin pigmentation [1]. The gingival pigmentation is formed by melanoblasts interlacing melanin granules between cells at the base of the gingival epithelium. The pigmentation degree differs depending on the melanoblastic interactions of each individual [1].

Gingival hyperpigmentation is a deep colour of the gingiva related to several exogenous and endogenous elements. There are many causes for pigmentation, such as drugs, genetics, endocrine disturbances, some syndromes like Peutz-Jeghers syndrome, and also some habits such as smoking that can motivate melanin pigmentation. It is commonly observed at the anterior labial gingiva and can be seen in females more than in males [2]. It can be noticed in dark-skinned individuals, where there is an increase in melanin production as a result of genetic hyperactivity of skin and mucosal melanocytes [3]. In previous reports, no significant difference has been observed between light-skinned, dark-skinned, and black individuals with regard to the distribution of melanocyte density [4]. In contrast, dark-skinned individuals have uniformly high melanocyte reactivity, whereas, in light-skinned individuals, melanocyte reactivity is highly variable [5].

Several procedures for gingival depigmentation (GD) have been authenticated, such as scalpel scraping, bur abrasion, electrosurgery, cryotherapy, and lasers [6]. All of these techniques involve the elimination of the epithelium layer along with the underlying connective tissue to regenerate a new gingival epithelium without melanin. Also, some researchers studied the role of ascorbic acid in the management of gingival melanin pigmentation [6-8]. The technique selection should primarily depend on the dentist’s clinical experience and patient preferences [9,10].

However, the microneedling (MN) technique is a technique of repetitive punctures, which is considered a collagen induction therapy. It has been extensively used as a dermatological treatment modality over the last several years due to its simplicity, cost-effectiveness, good tolerance, and ability to offer both cosmetic and therapeutic advantages [11]. It is a hybrid approach between transdermal patches and hypodermic needling, creating physical trauma that disrupts the stratum corneum layer. MN prompts the cascade of wound healing by forming small holes known as micro-conduits with minimal damage to the epidermis, which contribute to the rapid absorption of topical medications within the layer of the stratum corneum [11]. This successively causes the enhancement of growth factors that generate collagen and elastin production in the papillary layer [12].

A range of MN types has been introduced to manage scarring, wrinkles, pigmentation disorders, and hyperhidrosis [13,14]. Clinical reports have integrated MN into the management of pigmentation disorders in darker skin types, involving vitiligo, melasma, and periorbital hyperpigmentation [12].

Furthermore, vitamin C, a water-soluble antioxidant referred to as ascorbic acid (AA), is vital to the growth of strong bones, teeth, gums, ligaments, and blood vessels and is involved in important functions. Also, the anti-pigmentation properties of vitamin C are evident. It interacts with copper and inhibits the tyrosinase enzyme's action, thus decreasing melanin formation [15,16].

Published studies that tested the efficacy of the MN technique in the treatment of skin pigmentation were very limited. However, our study is the first to document the effect of this method on gingival mucosa. Hence, our aim of the study is to evaluate the efficacy of the MN approach with a combination of vitamin C in the management of gingival hyperpigmentation.

## Materials and methods

**Figure 1:**
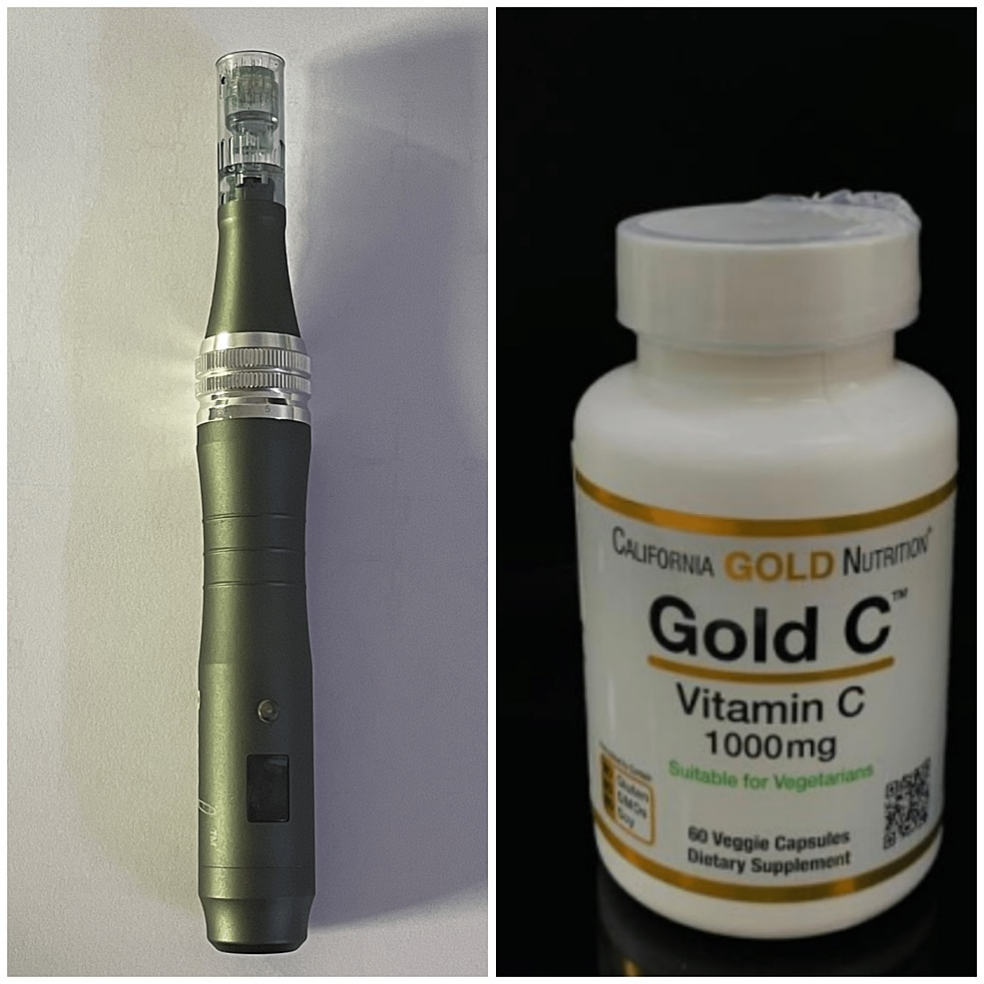
A Dermapen device and topical vitamin C (1000 mg) were the materials used in the microneedling technique.

In our study, we used ascorbic acid powder (1000 mg) and a Dermapen, which is an instrument that resembles a pen with a handle and needles and is powered by a rechargeable battery. The needle tip has 12-24 needles arranged in rows. There are six-speed modes where the highest speed mode is 700 cycles/min while the lowest speed mode is 412 cycles/min. Its handpiece allows the physician to treat areas in any desired direction; needle length is adjustable with the help of guides; and needle tips are disposable, which means that the same handpiece with a new needle tip and guide can be used on different patients (Figure 1).

Recruitment of Subjects

**Figure 2:**
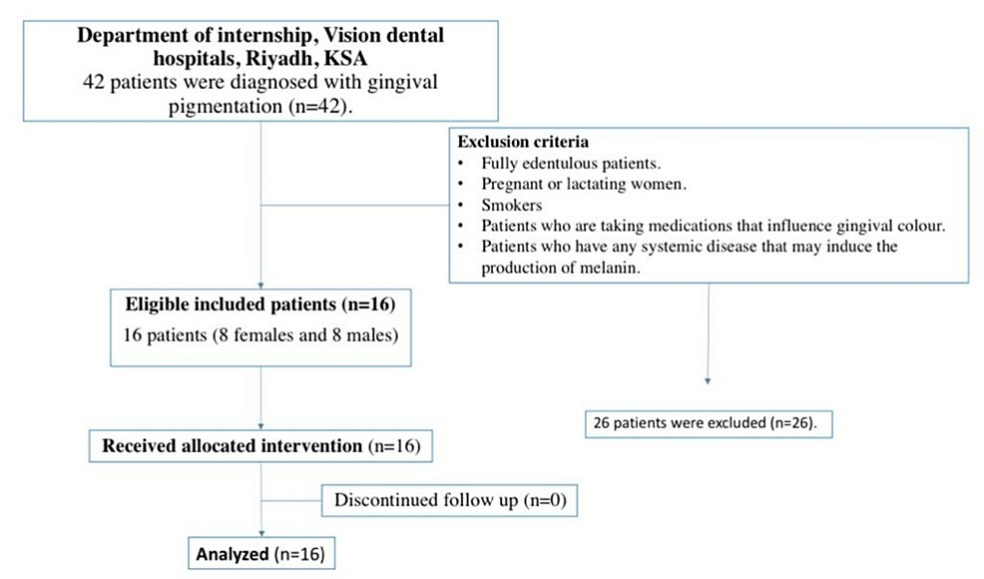
Consort flow diagram of patient selection

Ethical clearance was obtained with IRB#00010037 and reference approval number visi.dent-2021018, prior to the start of our study. The study was performed in accordance with relevant guidelines and regulations. Written informed consent was provided to each patient who decided to contribute to the study. The study population consisted of 16 out of 42 patients who met the inclusion and exclusion criteria. The included patients ranged in age from 17-35 years old; eight women and eight men were selected from the outpatient internship department at Vision Dental Hospital, Riyadh, KSA (Figure 2). They were clinically diagnosed with pigmented gingiva according to the new classification of periodontal and peri-implant diseases in 2017 [17].

Inclusion criteria included health-conscious patients between the ages of 14 and 50 with no previous treatment that affected the gingival health or colour. Individuals with mild-to-severe anterior melanin gingival pigmentation according to Dummet's classification were involved [18]. The exclusion criteria included fully edentulous patients, pregnant or lactating women, smokers, and patients who were taking medications that influenced gingival colour or had any systemic disease that may induce the production of melanin.

**Figure 3:**
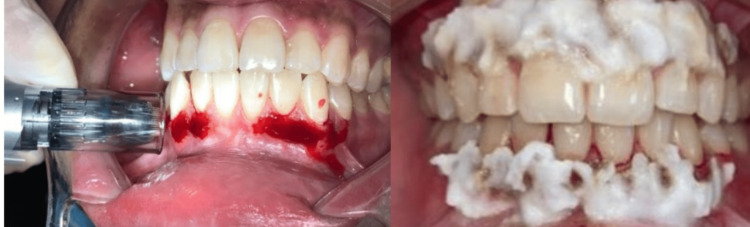
Microneedling technique A Dermapen device was used to create the microholes in the gingiva that will receive the topical application of ascorbic acid powder.

All participants were subjected to professional scaling and oral hygiene instructions before treatment. Under perfectly aseptic conditions and infiltration anaesthesia, the pigmented gingival epithelium from the right first premolar to the left first premolar received the MN technique using the Dermapen with needles of 1.5 mm depth. After ensuring the observation of the bleeding points on all affected gingiva, topical AA powder (1000 mg/ml) mixed with saline was applied to the gingival mucosa for 10 minutes as shown in Figure 3. The treated area was left without dressing. The patients were instructed to refrain from drinking acidic or hot beverages for 24 hours and not brush for one day to avoid any mechanical trauma to the gingiva. It was not necessary to prescribe mouthwash or medications following the procedure. The same procedure for the MN and AA applications was performed after two weeks. The photos were taken before, during, and at follow-up appointments after a month.

Clinical Assessment

Clinical evaluations included documented patient age and gender, medical and dental histories, oral care methods, and extra- and intra-oral examinations.

Pigmentation intensity scores (PIS) were assessed by the Dummett oral pigmentation index (DOPI) [18]. Preoperative and postoperative observations of melanocytic stains were recorded, giving scores from one to four: one for no observed pigmentation (pink gingiva); two for mildly observed pigmented gingiva with a light brown colour; three for moderately observed pigmentation with a medium brown or pink-brown mixed colour; and four for heavily observed pigmentation with a deep brown or blue-black colour.

Pigmentation extension scores (PES) were assessed by the Hedin melanin index (HMI) [19], where scores were recorded from 0 to four: 0 for no clinically evident pigmentation; one for one or two solitary pigmented units in the interdental papillae; two for three or more pigmentation units in the papillary gingival without a continuous ribbon pattern; three for more than or equal to one short continuous ribbon of pigmentation; and four for a continuous ribbon pattern involving the area between canines.

Follow-Up and Tissue Re-evaluation

Each patient was reviewed at three points in time: before treatment (baseline), two weeks after treatment, and one month after treatment.

Statistical Analysis

Data analysis was performed using IBM Statistical Package for Social Sciences (SPSS) version 26 (IBM Corp., Armonk, NY, USA), and a p-value of < 0.05 was considered significant. A paired T-test was done to evaluate pre- and post-treatment scores.

## Results

**Figure 4:**
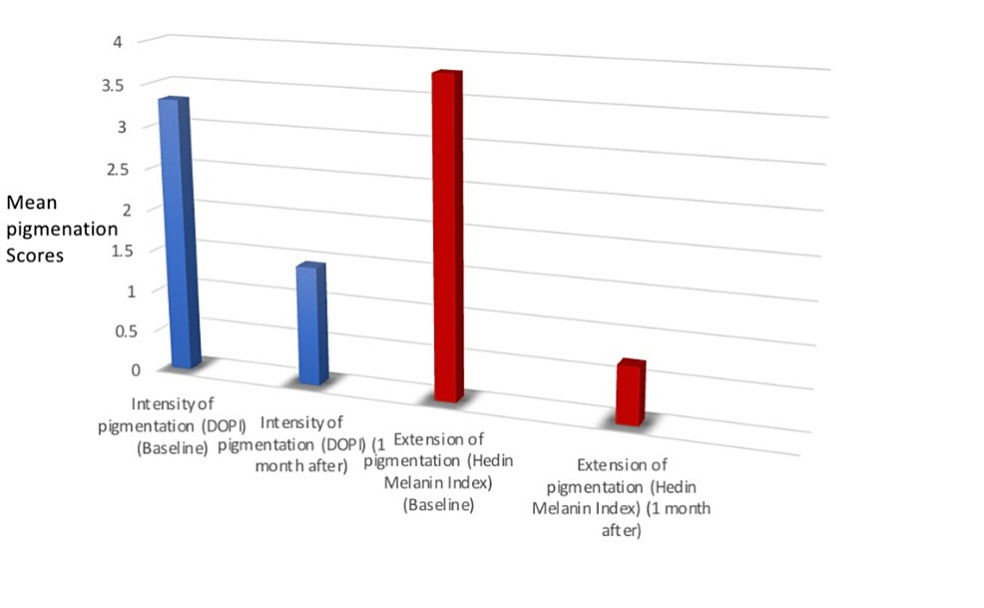
A graph of the pigmentation scores Comparison between pigmentation scores before and after the microneedling technique

Sixteen participants contributed to our study. The mean age of eligible participants was 24.8 ± 4.73 years. Fifty percent were men (n=8) and 50% were women (n=8). The mean DOPI score in the baseline and last session (after one month) was 3.31 ± 0.60 and 1.44 ± 0.51 retrospectively. On the other hand, the mean HMI score in the baseline and last session (after one month) was 3.81 ± 0.40 and 0.69 ± 0.70 respectively (Table 1 and Figure 4). Analysis using paired T-tests showed a statistically significant lower DOPI score post-treatment (mean difference 1.8 ± 0.7, 95% confidence interval (CI): 0.17-1.49, p ≤0.001). Similarly, the HMI score was lowered post-treatment with statistical significance (mean difference 3.1 ± 0.7, 95% CI: 2.74-3.50, p ≤0.001).

**Table 1:** Comparison between parameters (DOPI) & (HMI) before and after the treatment DOPI: Dummett oral pigmentation index; HMI: Hedin melanin index

Parameter	Score	Frequency (%)	Mean ± SD
Intensity of pigmentation (DOPI) (baseline)	1	0 (0)	3.31 ± 0.6
	2	1 (6)	
	3	9 (56)	
	4	6 (38)	
Intensity of pigmentation (DOPI) (one month after)	1	9 (56)	1.44 ± 0.51
	2	7 (44)	
	3	0 (0)	
	4	0 (0)	
Extension of pigmentation (Hedin melanin index) (baseline)	0	0 (0)	3.81 ± 0.40
	1	0 (0)	
	2	0 (0)	
	3	3 (19)	
	4	13 (81)	
Extension of pigmentation (Hedin melanin index) (one month after)	0	7 (44)	0.69 ± 0.70
	1	7 (44)	
	2	2 (12)	
	3	0 (0)	
	4	0 (0)	

**Figure 5:**
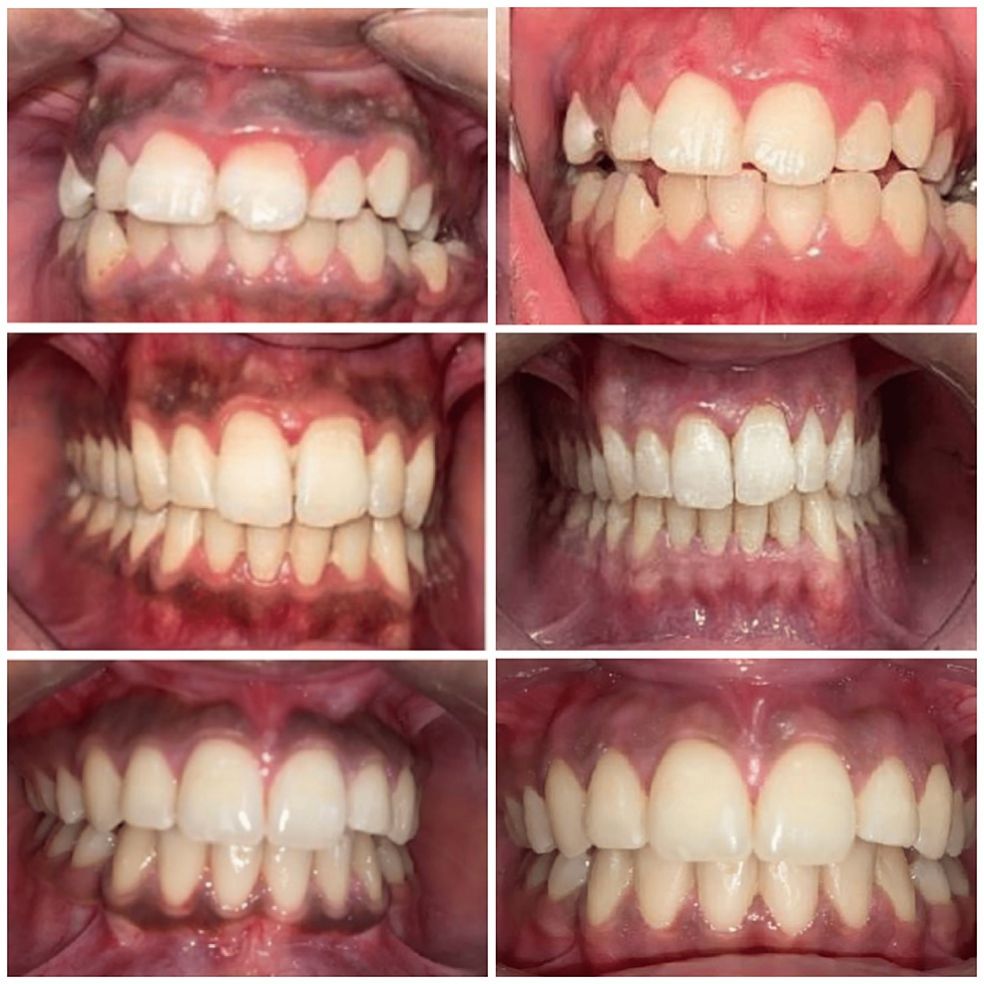
Preoperative and postoperative pictures

All study samples exhibited increased volume, altered texture, and changed colour one day after the procedure, indicating mild tissue inflammation without any systemic allergic reaction. There were no reports of pain or tenderness; only discomfort was experienced in most of the cases. Generally, healing was normal and satisfactory, and all patients achieved excellent aesthetic results with a reduction in both indices. Seven out of the 16 patients showed complete depigmentation of the gingiva, while nine patients displayed a reduction in their indices. Samples of some photographs of treated cases before and after treatment are shown in Figure 5.

## Discussion

Even though GP demonstrates no medical concerns, it is common for patients to seek cosmetic treatment for their "black gums." GD is a periodontal plastic procedure that involves the removal or reduction of gingival hyperpigmentation by a variety of techniques such as scalpel scraping, bur abrasion, free gingival grafting, electrosurgery, cryosurgery, and lasers [20]. In this study, we achieved GD using a novel technique using MN and topical AA. The procedure is minimally invasive, well-tolerated, safe, not expensive, and less time-consuming.

Among the various MN devices, the derma roller and the Dermapen are the most popular. Unlike other MN devices, the Dermapen is the only device that can be applied intraorally by virtue of its small size and interchangeable head, offering convenient application inside the oral cavity and allowing its use for a larger number of patients. This device is a wireless electric device with automated features that enables the operator to adjust speeds, pressures, and penetration depths, reducing the possibility of operator-associated risk factors.

During the procedure, a precise bleeding pattern triggered by MN must be performed for optimal results [21]. This creates microholes in the connective tissues that facilitate the penetration of the therapeutic medication, stimulating new collagen production and affecting melanocytes [11].

We used topical AA as a therapeutic medication; it reduces melanogenesis directly and promotes depigmentation within cells. Melanin is a reservoir for reactive oxygen species (ROS), copper (Cu), and calcium (Ca). After AA enters the target tissue, it binds to melanin, causing a depletion of the ROS, Cu, and Ca, resulting in a reduction in melanin production [22]. As such, AA affects the function rather than the number of melanocytes, as opposed to other approaches that destroy melanocytes [22].

In our study, we established excellent aesthetic outcomes that are compatible with a case report that was done by Mostafa D, who applied AA to gingival mucosa using the MN technique, resulting in complete gingival depigmentation that lasted for six months [7]. Also, these results were in agreement with the clinical trial that was established by Yussif et al., who injected 1-1.5 ml of the AA (200-300 mg) intraepithelially in the gingival mucosa of 40 patients and identified a statistically significant reduction in the incidence of pigmentation and area of pigmentation with a mean of 0.737 [8]. In 2019, Yussif et al. compared injectable AA to conventional scalpel depigmentation on thirty patients and concluded that injecting AA into pigmented cells yielded nearly equivalent and comparable results [23].

Additionally, our results were in agreement with those obtained by Shimada et al., who administered topical vitamin C gel to the gingival tissues and stated that it hindered melanin pigmentation [6]. In contrast, El-Mofty et al. conducted a comparative, random study on 20 patients, where 10 patients received intra-mucosal injections of AA and the other group received topically applied ascorbic acid gel. They reported that the mean area fraction of melanin-forming cells was significantly reduced in both groups, but the effect size was less in the group of topically applied AA gel (r=0.886) than intra-mucosal injections (r=0.797) [24].

However, these admirable aesthetic results were visible to the patients within a few days, unlike any other depigmentation procedure (Figure 3). Healing takes seven to 10 days after scalpel depigmentation, whereas healing after other procedures takes more than two weeks [3, 25]. Furthermore, a dressing is not required because the formed microholes heal quickly without perfusing bleeding, unlike with invasive scalpel or bur depigmentation, where gingival tissues should be covered by a periodontal dressing since they leave denuded connective tissues that heal via secondary intention [9]. Alternatively, GD can be achieved with a free gingival graft, but this procedure requires a second donor site and its results are not aesthetically appealing [26].

MN is less expensive and poses fewer complications than other minimally invasive procedures. While lasers are fast, painless, and effective in terms of hemostasis and decontamination effects, they are also expensive and sophisticated, and they may delay healing for up to one to two weeks [27,28]. Whereas, electrosurgery can cause excessive heat accumulation as well as unintended tissue destruction [29], while cryosurgery can lead to swelling and increased soft tissue destruction as well as difficulty in controlling the depth and duration of freezing [25]. However, the longevity of GD depends on the treatment methods and genetic and ethnic factors [25]. Surgical techniques have shown early recurrence after 15 days to six months [30-32], while lower recurrence results were documented after 12-48 months when using laser and cryosurgery for GD [32-34]. On the other hand, recurrence of GP using ascorbic acid was reported in varying studies after six to nine months [7,8,35].

A wide variety of gingival hyperpigmentation treatments are available; they should be chosen based on clinical experience, affordability, and personal preferences. However, as our study covered only these limited numbers of cases with short follow-up periods, additional studies involving a larger sample size with long follow-up periods are needed to verify our findings.

## Conclusions

In our research, we have applied a new method of combining MN and topical AA that showed promising results, creating new prospects in treating GP. In spite of the fact that our method is a safe, simple, and cost-effective GD method that requires no professional experience, it cannot be used at home. Generally, it is crucial to perform GD techniques carefully in order to prevent recession of the gingiva, uneven healing, and tissue destruction.
